# Book Review: We Can’t Say We Didn’t Know

**DOI:** 10.1177/1329878X20949947

**Published:** 2020-11

**Authors:** Scott Poynting

‘#wecantsaywedidntknow’ was the hashtag under which, in 2016, Sophie McNeill tweeted news from her informants under siege in Aleppo (p. 170), where hospitals were knowingly bombarded by Syrian and Russian military, and masses of civilians, many of them children, indiscriminately killed. McNeill is an investigative reporter with the ABC, now with the *Four Corners* programme. From 2015 to 2018, she was the ABC’s Middle East correspondent, and *We Can’t Say We Didn’t Know* enlarges mainly upon her reports from that time. McNeill was previously a member of the SBS *Dateline* team from 2005 to 2010, and had reported for them from Lebanon, Iraq, Egypt, Israel, Jordan, Afghanistan and Pakistan, among other countries, winning two Walkley awards over this period, and later a third during her ABC Middle East posting. The book, McNeill’s first, includes cutting-edge material from Syria, Yemen, Gaza, the West Bank, Iraq, Jordan and elsewhere.

It makes a harrowing read. Much of the reporting covers state crime, war crimes, crimes against humanity: deliberate starving of civilians under siege, denial of medical treatment, purposeful targeting of hospitals and medics, disappearances, torture, political executions, unlawful weapons, indiscriminate killing of civilians. It is not dispassionate – how could it be? – but reported facts are rigorously corroborated and related accounts are supported with abundant evidence. In 2015, McNeill relayed reports from Madaya near Damascus, where children were starving to death under siege by Assad’s forces, with food and medical relief from the United Nations (UN) and the Red Cross blockaded. All of these stories in the book are told using the voices, the experiences, of those suffering the atrocities, rendering the accounts real, human and accessible. McNeill paces and sequences the stories cleverly; she writes well, and with warmth and deep humanity. Sometimes with anguish, as when those voices say to stop filming, they have had enough, it does not help. As Aleppo fell, besieged residents told the colleague providing video footage to McNeill, ‘For five years we’ve been seen killed. What has changed? Now people here just want to die in peace’. The author confesses, ‘I left ashamed to admit they were right’ (p. 190). And yet,

to look away was criminal. If this was the world we had created, where war crimes were allowed to be carried out live, day after day with no consequence, then we were – at the very least – required to watch and recognise the full cost of our inaction. (p. 189)

The ABC’s foreign desk back in Australia did not always agree.

The ‘Dispatches from an age of impunity’ subtitled on the book’s cover apply equally to state crime and breaches of international law in Gaza and the West Bank, in Yemen, and other places covered. The work is brave, principled, honest and impeccably professional, and based on highly talented and committed investigation and story-telling.

The book’s opening epigraph presents verses from Palestinian politician and poet, Tawfiq Ziad’s, ‘Our Country is a Graveyard’, ‘. . . you have planted bullets in our heads, and organized massacres/Gentlemen, nothing passes like that without account/All that you have done to our people is registered in notebooks’. Sophie McNeill’s notebooks bear very powerful witness.

Scott Poynting *Charles Sturt University*

